# YOLO-GW: Quickly and Accurately Detecting Pedestrians in a Foggy Traffic Environment

**DOI:** 10.3390/s23125539

**Published:** 2023-06-13

**Authors:** Xinchao Liu, Yier Lin

**Affiliations:** College of Mechanical Engineering, Tianjin University of Science and Technology, Tianjin 300222, China; liuxinchao@mail.tust.edu.cn

**Keywords:** YOLO-GW, pedestrian detection, foggy weather, dark channel de-fogging, model pruning, ECA module

## Abstract

In practice, the object detection algorithm is limited by a complex detection environment, hardware costs, computing power, and chip running memory. The performance of the detector will be greatly reduced during operation. Determining how to realize real-time, fast, and high-precision pedestrian recognition in a foggy traffic environment is a very challenging problem. To solve this problem, the dark channel de-fogging algorithm is added to the basis of the YOLOv7 algorithm, which effectively improves the de-fogging efficiency of the dark channel through the methods of down-sampling and up-sampling. In order to further improve the accuracy of the YOLOv7 object detection algorithm, the ECA module and a detection head are added to the network to improve object classification and regression. Moreover, an 864 × 864 network input size is used for model training to improve the accuracy of the object detection algorithm for pedestrian recognition. Then the combined pruning strategy was used to improve the optimized YOLOv7 detection model, and finally, the optimization algorithm YOLO-GW was obtained. Compared with YOLOv7 object detection, YOLO-GW increased Frames Per Second (FPS) by 63.08%, mean Average Precision (mAP) increased by 9.06%, parameters decreased by 97.66%, and volume decreased by 96.36%. Smaller training parameters and model space make it possible for the YOLO-GW target detection algorithm to be deployed on the chip. Through analysis and comparison of experimental data, it is concluded that YOLO-GW is more suitable for pedestrian detection in a fog environment than YOLOv7.

## 1. Introduction

With the rapid development of driverless cars in recent years, they have been equipped with a visual perception system composed of cameras and embedded devices. The visual perception function primarily includes scene analysis and environment perception, among which scene analysis plays a dominant role. The scene analysis function primarily includes three research tasks: Object localization, image classification, and semantic segmentation. Visual object detection is the basic function module in autonomous vehicle scene analysis, and it is also the module that people are keen to study. The safety of autonomous vehicles largely depends on the object detection of the obstacles in front. However, the complex and changeable autonomous vehicle traffic scenes pose great challenges to the object detection algorithm, primarily including (1) the influence of bad weather such as haze and illumination on the accuracy of object detection; (2) how to maintain high-precision detection performance in the case of real-time detection; and (3) chip computing power and operating space limitations. When the object detection algorithm fails to extract complete feature information in time, there will be category errors in object recognition. Fog or haze has a serious impact on the recognition of traffic road information. The object detection algorithm, which is helpful in solving these problems, is the primary object detection algorithm based on deep learning, which has been popular in recent years.

Object detection algorithms based on deep learning can be divided into four schemes: (1) For object detection algorithms of region proposals, the typical networks are R-CNN [1] and Faster R-CNN [2]; (2) object detection algorithms based on regression, the typical representatives of which are YOLO [3] and SSD [4]; (3) object detection and recognition algorithms based on search, such as AttentionNet [5]; and (4) algorithms based on Anchor-free, such as CornerNet [6], CenterNet [7], FCOS [8], and so on. The above four schemes have laid the foundation for the subsequent research. Zou [9] used convolutional neural networks to extract high-level features from sub-images and perform local feature weights and used recursive neural networks to classify feature sequences in the appropriate order. Wang [10] replaced all BN layers with SN layers, and ResNet-101 [11] was used as the backbone network to improve the CSP of the pedestrian detector [12]. Song et al. [13] detected pedestrians in various environments based on deep learning and multi-spectral feature fusion, which was robust. All of the above studies have improved the performance of target detection algorithms to varying degrees. These optimization strategies show excellent detection effects in the detection of the road environment in good weather conditions, but there are serious omissions and false detection in bad-haze weather conditions. In order to better detect pedestrians in a heavy-haze road environment, we need to develop more reasonable detection strategies.

Aiming at the problem of de-fogging in a foggy environment, Fan et al. [14] proposed a new image de-fogging algorithm based on sparse domain training. It learns the fog-free sparse features in good weather, reconstructs the fog image, and uses a nonlinear stretching method to enhance the brightness of the image. However, it is not good at feature extraction, which leads to the problems of local defogging and color features missing in the foggy picture. Yang et al. [15] proposed a depth perception method to predict depth images based on the generated adversarial network structure and then provided depth features for fog removal in the network framework, which was of great help to the removal of haze in the environment. The disadvantage was that the effect of removing distant fog in the image was poor. The above studies have made some improvements regarding the problem of fog removal, but there is still a need for further optimization. Han [16] proposed an improved RCNN target detection model aimed at the detection problem of the target detection algorithm in a foggy-day environment. By optimizing the feature pyramid, shallow feature information is combined into a deep network to improve the extraction of information features, and an optimization module is added to enhance the robustness. This model has rich background information. However, there are problems of missing detection and false detection in the results of detecting foggy images. Cao et al. [17] enhanced the loss function of the SSD model, adopted a weighted mask for network training, and strengthened the loss function. Thus, the anti-jamming ability of the target detection algorithm is improved in bad weather, but its detection efficiency is not so good. Qin et al. [18] proposed a lightweight ThunderNet detector. A relatively efficient RPN and detection head are designed, and a spatial attention module and an enhancement module are added to improve the feature representation of the image. The ThunderNet detector enhances the detection accuracy and reduces the detection efficiency compared with the single-stage detector. All of the above-mentioned research studies improve the performance of the target detection algorithm to varying degrees. However, the rationality of balance detection efficiency and accuracy needs to be further improved. Otherwise, when the pedestrian in the traffic environment is blocked by haze, the object detection algorithm cannot extract the complete feature information effectively and quickly, resulting in the omission or false detection of object recognition, and eventually the occurrence of traffic accidents.

In order to solve these problems, four improvement measures were developed in this experiment to solve the problem of real-time detection of pedestrians on foggy roads by driverless cars. The dark channel defogging algorithm with a good defogging effect is adopted for defogging treatment. A large-scale, diversified, and robust BDD100K [19] dataset is selected to enhance pedestrian detection in urban traffic scenes. An excellent dataset is particularly important for the learning of the detection model. Compared with other pedestrian datasets, the BDD100K [19] dataset is more suitable for the learning of the urban traffic scene detection model. Secondly, the detection head of YOLOv7 was added to make it fit for the detection of more object sizes, so as to improve the detection ability of pedestrian objects. To solve the problem of computing resource consumption of the algorithm, a reasonable pruning strategy was developed to ensure the algorithm meets the requirements of vehicle chip hardware resources. Developers design the network in the training process through unsupervised learning of the weight of the network layer, to train the importance of each part. Therefore, in the deep learning algorithm, not all learned weights are equally important, and there are some redundant or less important network modules. On the premise of keeping the detection accuracy constant, the detection speed can be improved by pruning these network modules. Based on this idea, combined with the research work of Liu [20], Ren [21], Wang [22], and Ye [23], a combination pruning strategy was proposed. Combined with the pruning strategy, sparse L1 regularization was applied to channel scale factors to improve channel level sparsity and trim channels with smaller scale factors. In addition, adding layer pruning to channel pruning and evaluating the convolutional layer will trim the layer with the lowest scaling factor mean, resulting in a “refined” detector. The pruned network is then fine-tuned to take full advantage of the potential performance of the pruned network model. Compared with the original model YOLOv7, the trainable parameters of model YOLOv7^+^-87% are reduced by 97.66%, the model space is reduced by 96.36%, and the reasoning speed is 432.30% faster.

## 2. Related Work

### 2.1. Model Is Introduced

The traditional object detector extracts image features by a sliding window, but its disadvantage is that the efficiency of object appearance processing is low. After R-CNN [1] was proposed, its significant advantages aroused extensive research interest and promoted the development of a depth object detector. In recent years, many excellent depth object detectors have emerged, including outstanding SPP-net [24], Fast R-CNN [2], R-FCN [25], R-CNN [1], SSD [4], YOLO [3], YOLOv2 [26], YOLOv3 [27], YOLOv4 [28], YOLOv5 [29], YOLOv6 [30], YOLOv7 [31], etc. These object detectors can be divided into two categories: One is a two-stage object detector represented by R-CNN and Fast R-CNN, which consists of three modules: A regional recommendation module, a backbone network, and a detection head. Firstly, the region suggestion module generates a large number of suggestions that may contain regions of interest. The detection head detects the categories of these suggestions and then performs position regression to precisely locate its objects. The two-stage object detector achieves high detection accuracy by a more precise region suggestion. While obtaining high-quality detection accuracy, its reasoning process needs to consume huge computing power and running space, resulting in relatively slow object detection speed. The other is the single-stage object detector represented by the YOLO series and SSD. K prior boxes are set in each position of the feature map, and each prior box corresponds to a specific position instead of using similar regional recommendation network processing, which simplifies the calculation process and improves the detection efficiency. Therefore, the single-stage object detector runs faster than the two-stage object detector. In the single-stage detector, the YOLOv7 object detection algorithm has better detection speed and higher detection accuracy, so in this study, we chose the YOLOv7 object detection algorithm as the basic network of this experiment.

In order to reduce the consumption of hardware resources by the deep learning algorithm, a combination pruning strategy is verified by experiments. YOLOv7 integrates pruning strategies to learn a more efficient object detection model, namely YOLOv7*. Then, the fog removal algorithm is used to process each frame of foggy images read by the camera so as to improve the detection effect of the object detection algorithm on foggy days. Determining how to improve pedestrian detection in a foggy environment on the premise of improving the accuracy and speed of object detection is difficult in the research field. In combination with the above strategies, this experiment adopted a rich and multivariate BDD100K dataset to enrich the road pedestrian features in the model. The detection header is added to the YOLOv7 object detection network to improve the detection accuracy of small objects. The pruning strategy is integrated to eliminate the redundant parts in the training model. Finally, in front of the image input network model, the pedestrian information in a foggy environment is de-fogged.

### 2.2. Model Compression

On the vehicle chip with limited resources, the combination pruning strategy is an effective way to reduce the resource consumption of the object detection algorithm. At present, popular model compression methods include knowledge extraction [32], dynamic calculation [33], parameter quantization [34], model pruning [20,23], etc. The model compression method adopted in this study is model pruning.

**Figure 1:**
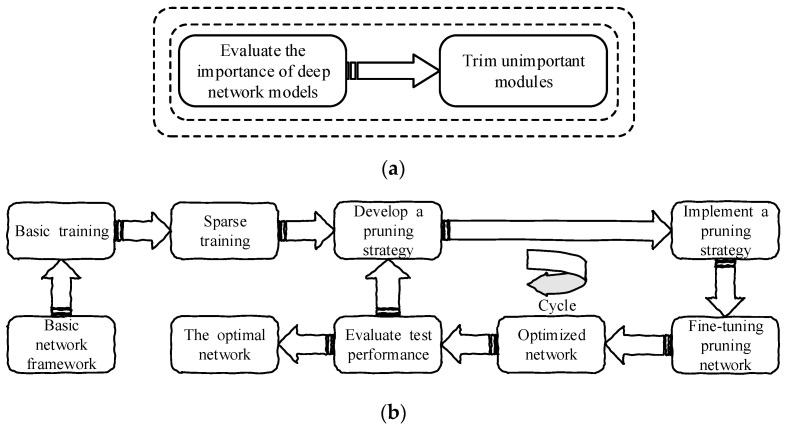
(**a**) Develop a pruning strategy; (**b**) pruning flow chart; iteration steps: (1) Evaluate the importance of sparse training algorithm model and formulate pruning strategy; (2) fine-tune the pruned model to fully improve the potential algorithm performance; (3) after evaluating the performance of the optimized model, if the performance does not meet the optimal requirements, the pruning deployment is performed again to reach the optimal network model.

Figure 1 shows the entire pruning process. In the pruning process of the network model, the neurons in the deep model and network model in the deep network are primarily pruned. The idea of structured pruning was adopted in this experiment [35]. Compared with weighted pruning, structured pruning makes it easier to store the pruned network model and improve its speed. The weighted pruning network model needs a special hardware and software library, which is difficult to implement in practice. The advantage of structured pruning is that it is easier to generate a regular and convenient network model. Researchers promote structured pruning through structured sparsity regularization and sparse training, which primarily includes sparsity and structured sparsity learning on channel scale factors. Liu et al. [20] proposed a simple and effective channel pruning method, called network model simplification. The scale factor in the normalized layer of batch processing is primarily used as the scaling factor of channel orientation, and channel sparsity is primarily used to train the scale factor of L1 regularization [21,22,23]. The depth model is improved by using the network slimming method, which significantly increases the computing speed and reduces the volume of the model. In this experiment, we used Liu’s [20] method to improve it as a coarse-grained depth model exploration method to seek a more efficient depth model detector.

## 3. YOLO-GW

**Figure 2:**
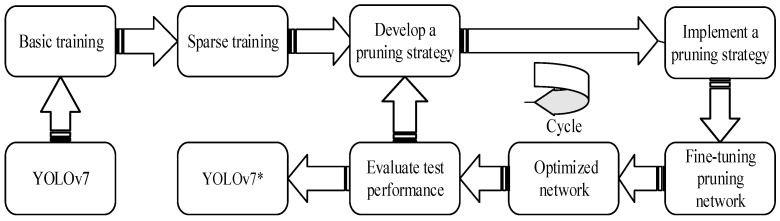
YOLOv7 was lightened by pruning strategy to obtain YOLOv7*. The pruning strategy is as follows: (1) YOLOv7 conducted basic training; (2) sparse training on the basis of basic training; (3) evaluate the importance of sparse training model and develop pruning iteration strategy; (4) fine-tune the pruning model to fully improve the potential performance of the algorithm; (5) after evaluating the performance of the optimized model, if the performance does not meet the optimal requirements, pruning deployment will be carried out again to achieve the optimal network model.

This experiment is a model pruning based on YOLOv7, and the optimal network YOLOv7* can be obtained through Figure 2.

**Figure 3:**
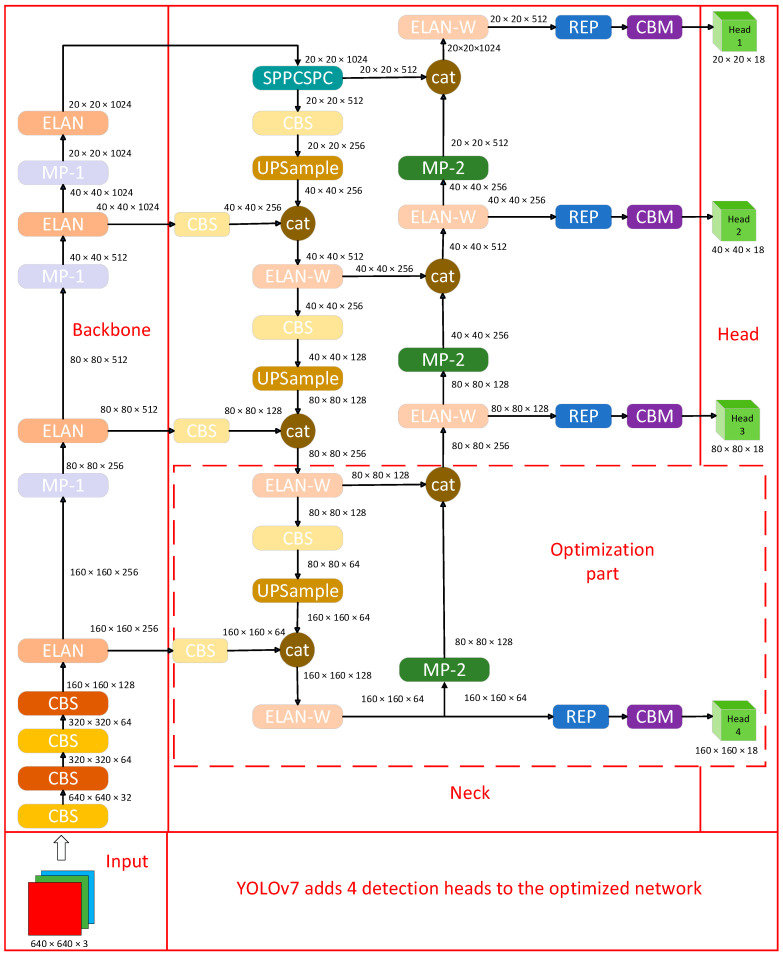
The optimized network structure of YOLOv7 is primarily to add a detection head on the basis of the original network of YOLOv7, as shown by the dotted line in the figure.

**Figure 4:**
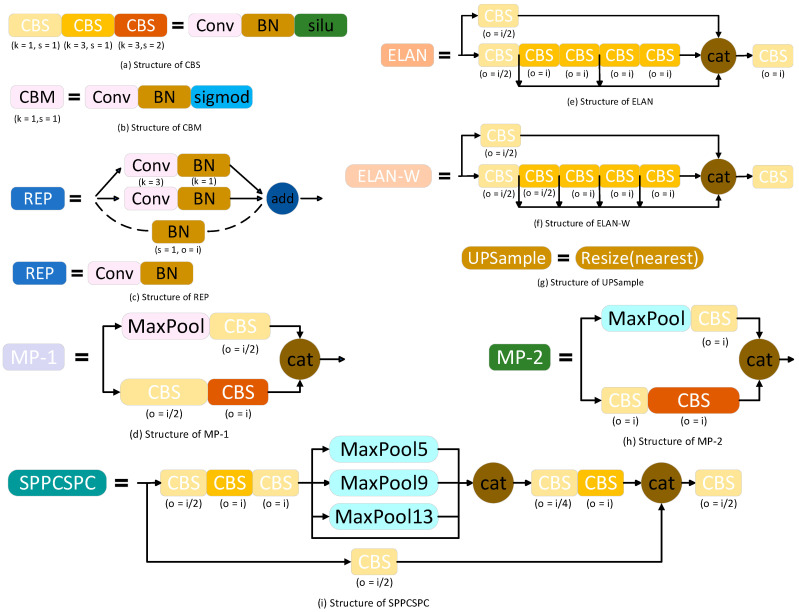
YOLOv7 optimized network structure decomposition.

There is a big difference between the YOLOv7 algorithm and the current mainstream object detection algorithm. In addition to the optimization of the network architecture, the YOLOv7 algorithm also adds some optimization methods in the process of network training. The optimization of network architecture and training mode enables YOLOv7 to improve the detection accuracy of the object without increasing the calculation cost. The optimized network structure of YOLOv7 shows the optimization of the backbone part in Figure 3, and the modules of the network framework are decomposed in Figure 4: The CBS in (a) is composed of a convolutional layer, a BN layer, and a Silu activation function. The CBS has three colors of modules, K represents the convolution kernel, S represents the step size, and the colors represent the three types of asynchronous lengths and the convolution kernel CBS module. The CBM module in (b) is composed of a convolutional layer, a BN layer, and a sigmoid function. The convolution kernel is 1 × 1 and the step size is 1. The REP module in (c) is divided into two types. One is the training module, which is composed of three branches, which are 3 × 3, 1 × 1, and the third layer is without convolution operation. The other is the reasoning module, which contains 3 × 3 convolution with a step size of 1. The MP-1 module in (d) has two branches, which are primarily used for down-sampling. The first branch passes through Maxpool and then through a 1 × 1 convolution. The second branch is a convolution of 1 × 1, a convolution of 3 × 3 with a step size of 2, and then the results of the two branches are added. The ELAN module in (e) has two branches: One after a 1 × 1 convolution, the second after a 1 × 1 convolution, followed by four 3 × 3 convolutions, and finally, the four features are superimposed together. The ELAN-W module in (f) differs from the ELAN module in (e) in that the ELAN-W module selects five outputs for superposition. The up-sample module in (g) uses the nearest neighbor interpolation as the up-sampling method. The MP-2 module in (h) has the same architecture as the MP-1 module in (d). The difference is that the step size of the convolutional block is different. The SPPCSPC module in (i) has two branches. The first part is the processing of the SPP module, and the second part is routine processing. Finally, the two parts are combined to reduce the amount of calculation by half, so that the speed becomes faster, and the accuracy will be improved.

This paper adds an ECA module [36] on the basis of the YOLOv7 algorithm to optimize the performance of the YOLOv7 algorithm. ECANet is a partial optimization on the basis of the SENet module and proposes a local cross-channel interaction strategy without dimensionality reduction, also known as an ECA module. This module adds few parameters but effectively improves the performance of the object detection algorithm. In addition, in order to enhance the detection performance of YOLOv7, three detection heads were expanded to four detection heads. The detection heads of YOLOv7 were primarily used for object classification and regression, and enhanced effectiveness features could be obtained in the backbone network and the FPN network. The increase in detection heads not only enhanced object classification and regression but also improved effective feature extraction.

Basic training: Set the training parameters of the main network and select the appropriate dataset for basic training.

Sparse training: The purpose of sparse training [37,38] is to facilitate the pruning of the channel and network layer by the pruning strategy in the later stage. In the experiment, a scaling factor is implanted in each channel for the convenience of channel pruning, and its absolute value is used to express the importance of the channel. In detail, the BN layer after each convolutional layer without a detection head is used to improve the generalization ability and achieve fast convergence. The BN layer uses small batch statistics to normalize internal activation, and the conversion of the BN layer is as follows:

(1) $$\overset{\wedge }{x}=\frac{x_{in}-\mu_{B}}{\sqrt{\delta_{B}^{2}+\varepsilon }};y_{out}=\gamma \overset{\wedge }{x}+\beta$$

where $x_{in}$ and $y_{out}$ represent the input and output characteristics of the BN layer, respectively; $\mu_{B}$ is the mean value of the small batch input; $\delta_{B}^{2}$ represents the variance of the small batch input; and γ and β represent trainable scales and displacements.

The trainable scaling factor in the BN layer is used to measure the importance of the network channel. In the experiment, the scaling factor is multiplied by the output of the channel to combine the training weight and scaling factor. After γ completes the regularization of L1 [39,40], channel sparse training is started to distinguish the unimportant channels from the important ones. According to Formula (2), the object of sparse training is given as

(2) $$L=T_{loss}+\eta \sum_{\gamma \in \Gamma }Χ\left(\gamma \right)$$

where η is used to balance $T_{loss}$ (representing the loss of normal training in the network) and Χ(•) (the penalty of scaling factor caused by sparsity), and X(γ)=|γ| is the regularization of L1. The subgradient method is adopted to optimize the penalty term of non-smooth L1.

Develop pruning strategy: The optimal pruning strategy is a pruning method combining layer pruning and channel pruning. It is primarily used to introduce a global threshold to limit the pruning degree of pruning after sparse training is completed. In order to reduce the destructive pruning model of the network, a local security threshold is adopted to prevent the over-pruning of the network model. Firstly, the pruning rate was adjusted by setting the global threshold $\overset{\cdot }{\gamma }$ to be ρ% of all γ . In order to avoid over-pruning, the local safety threshold ϕ was proposed, and ν% of all γ was set in the layer requiring pruning. When the scaling factor of the channel is less than the minimum values of $\overset{\cdot }{\gamma }$ and ϕ , it can be pruned to meet the requirements of pruning. If the scale factor of the whole layer is less than the threshold, in order to avoid the pruning of the whole layer, the channels with the largest scale factor in the layer are left. In addition, layer pruning is integrated on the basis of channel pruning. It is primarily used to evaluate the convolutional layer and then to rank the mean value of the scaling factors of each layer and cut out the corresponding part of the minimum value.

Fine-tuning the pruning network: After pruning the model, its performance will decline temporarily. Fine-tuning [41] is adopted to adjust the potential performance of the pruned model to the optimal level.

Evaluation of detection performance: The optimized new network is evaluated according to the evaluation index to determine whether the new network achieves the optimal detection performance. To achieve the optimal detection performance, we must stop pruning, that is, the optimal model YOLOv7*. The optimal detection performance is not achieved, and we need to trim again. In the process of pruning again, we need to prevent the occurrence of over-pruning, and the over-pruned model cannot be repaired.

**Figure 5:**
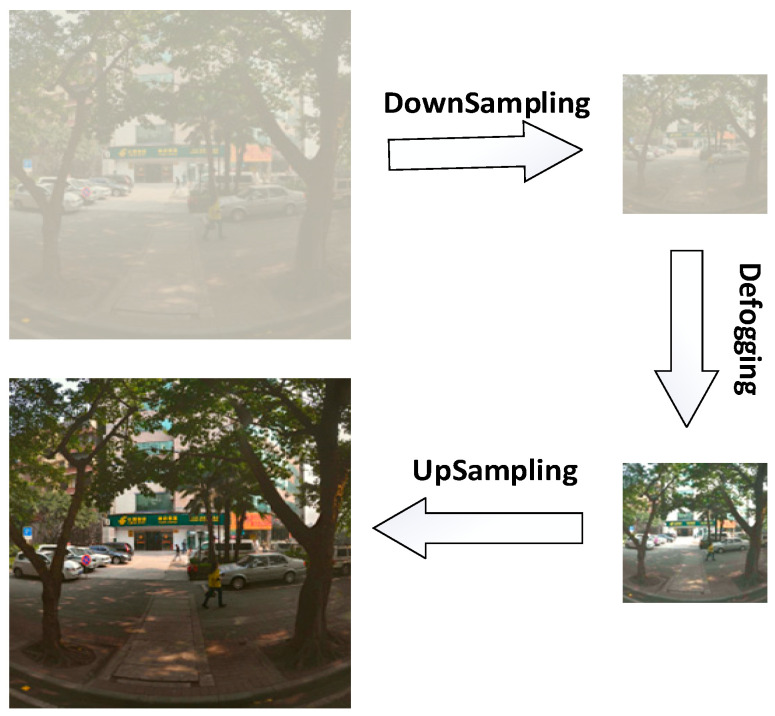
De-fogging strategy, in order to solve the defect of low efficiency of the dark channel defogging algorithm; the down-sampling and up-sampling modules are added to the strategy of the algorithm to improve the efficiency of the dark channel de-fogging algorithm.

The de-fogging strategy for a traffic environment: The dark channel de-fogging algorithm is selected in this experiment. The advantages of this algorithm are a good restoration effect and a simple application scenario, while the disadvantages are the low operating efficiency of the algorithm. In order to solve this problem, an optimization strategy of the defogging algorithm is formulated. As shown in Figure 5, we first execute Gaussian Smoothing in the original image with fog and then down-sample it 4 times. Then, the image after down-sampling is de-fogged in the dark channel [42]. At this time, the processed image is 1/256 of the original image area, and the de-fogging efficiency has been effectively improved. Then, the image without fog is up-sampled, and Gaussian Smoothing is conducted after up-sampling. The final image obtained meets our requirement of resolution. Finally, the processed image is input into the network for object recognition. YOLO-GW is an optimization algorithm based on YOLOv7 network architecture optimization, combined pruning, and de-fogging strategies.

## 4. Experimental

In order to verify the pedestrian detection performance of this algorithm in a foggy environment, the BDD100K dataset was selected and a combination pruning strategy was proposed to improve the effective depth object detection algorithm. On the basis of YOLOv7, YOLO-GW is further proposed, which not only satisfies the real-time detection in a foggy environment but also reduces the consumption of computing resources and operation space of hardware devices.

### 4.1. Experimental Environment

In order to conduct model training and performance testing, the experimental environment equipped with the algorithm is shown in Table 1:

### 4.2. Data Set

**Figure 6:**
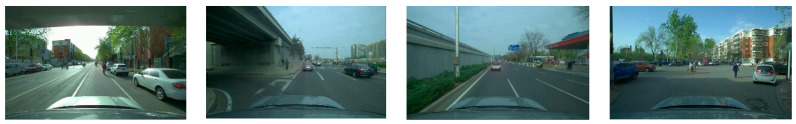
Self-made dataset sample.

The BDD100K [19] dataset is used in the experiment. This dataset has large-scale diversified information and covers a large amount of street information, which is particularly important for the robustness of the perception algorithm. The dataset contains 100,000 images of foggy, sunny, cloudy, rainy, snowy, cloudy, and day–night traffic conditions. The dataset contains 129,262 pedestrians, and each image contains approximately 1.3 pedestrians on average, among which 86,047 pictures contain pedestrians. Furthermore, 10,000 pedestrian-containing images are selected from all available images to form the experimental dataset. Specifically, the training set comprises 7000 pedestrian images, the validation set comprises 1500 pedestrian images, and the test set comprises 1500 pedestrian images. For visualization validation, 1000 self-constructed dataset images (which are not involved in training) are adopted. These images are of 640 × 400 pixels, as shown in Figure 6.

### 4.3. Training

First of all, general training was carried out on the selected basic network. Both YOLOv7 and YOLOv7**^+^**-87% were trained with a batch gradient descent. The pedestrian dataset of BDD100K was used for training, whereby 16 images were loaded into the network at a time, forward propagation was completed four times, and 100 times of iterative training was conducted for every four images. The initial learning rate was set at 0.0013, and the first six iterations were the warmup. The learning rate at 70% and 90% of the total iteration was multiplied by 0.1, respectively. The weight attenuation is 0.0005 and the momentum is 0.937. In the training, the algorithm will cut or fill the size of the dataset input into the network into the same size as the input picture set in the network. In order to better adapt to the dataset, the input network image is adjusted to 864 × 864, and the network input image size is set up close to the dataset’s size of pixels based on the optimization model of speed and accuracy of power. In this experiment, the weights trained by the researcher are used to initialize the selected network model.

Sparse training: The selected network first completes the basic training of 100 iterations, and then carries out the sparse training of 300 iterations [38,43]. According to the different learning rates set, we chose 0.0001, which had the best experimental effect out of the three penalty factors of 0.01, 0.001, and 0.0001, as the penalty factor for this training. During the sparse training, other parameters remained unchanged, and the setting was the same as that of basic training.

Fine-tuning: Improve potential detection performance by fine-tuning YOLOv7*-50%, YOLOv7*-86%, YOLOv7*-87%, YOLOv7*-88%, YOLOv7**^+^**-50%, YOLOv7**^+^**-86%, YOLOv7**^+^**-87%, and YOLOv7**^+^**-88%. These models are initialized with pretraining weights. In YOLOv7-N and YOLOv7-N, N is the pruning rate.

### 4.4. Evaluation Index

In this experiment, the following evaluation indexes were used to evaluate the effect of the pruning model: (1) Recall represents the rate of recall, (2) F1-score is the measurement index of the classification problem ranging from 1 to 0, (3) mAP indicates the mean Average Precision, (4) FPS is the number of inference frames per second, (5) parameter indicates the parameter size, and (6) volume is the model space. The above evaluation indexes are models to evaluate different pruning rates under the same dataset, experimental environment, and training parameters.

## 5. Experimental Results and Analysis

In Table 2, Table 3 and Table 4, 640 × 640 and 864 × 864 respectively represent the size of the network input picture set during the training of the model. The data are the evaluation index obtained from the training on the BDD100K pedestrian dataset. The performance of the basic model and the optimized model is evaluated through the analysis of the data. In YOLOv7-N, N is the pruning rate, and N is 50%, 86%, 87%, and 88%, respectively.

**Figure 7:**
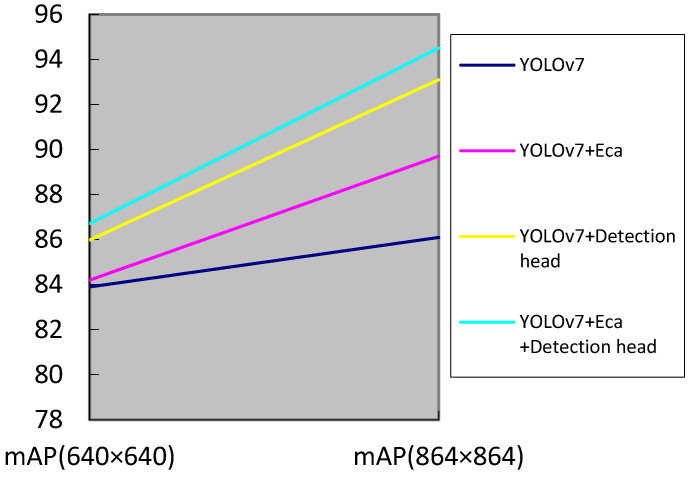
Comparison of mAP performance of ECA module and detection head module was added to YOLOv7 algorithm model.

In this experiment, YOLOv7 was selected as the basic network, but the performance of the original network could not meet the requirements of this experiment. After several tests, the ECA module was added to the YOLOv7 algorithm for optimization in this experiment. An efficient channel attention module could effectively improve the algorithm performance of YOLOv7 and only add a few parameters. In order to further improve the algorithm performance of YOLOv7, we changed the network structure of YOLOv7 with three detection heads into a network structure of four detection heads and carried out 864 × 864 large-scale training to further strengthen object classification and regression, as well as improve the effective feature extraction. As can be seen from Figure 7, mAP was significantly improved with the addition of ECA, a detection head, and large-scale network training (the advantage of setting the size of the network training image as 864 × 864 is to follow the principle that the higher the image resolution, the larger the object size and the simpler the feature extraction). Finally, these three optimization methods were added to the YOLOv7 network at the same time. As can be seen from Figure 7, mAP was further improved. As can be seen from Table 2, these methods not only improve the network accuracy but also reduce the reasoning speed of the network to some extent. The YOLOv7 network directly decreases from 65 frames per second to 22 frames per second. The target detection algorithm of 22 frames per second detection speed combined with the defogging strategy cannot quickly detect pedestrians in traffic scenarios. Therefore, we integrated a pruning strategy on the basis of YOLOv7 network optimization.

**Figure 8:**
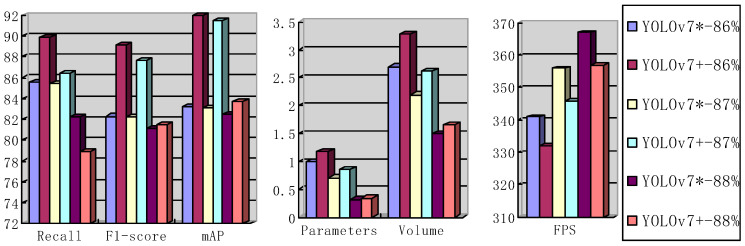
When the input image size was set to 864 × 864 for network training, 86%, 87%, and 88% pruned evaluation indexes were compared for the original network YOLOv7 and optimized network YOLOv7**^+^**, respectively.

Analysis of test effect: Table 3 lists several representative model evaluation indexes of YOLOv7 and YOLOv7**^+^.** Among them, YOLOv7**^+^** is an algorithm obtained through the optimization of the ECA module, detection head, and large-scale network training. There are six pruning models, namely YOLOv7*-86%, YOLOv7*-87%, YOLOv7*-88%, YOLOv7**^+^**-86%, YOLOv7**^+^**-87%, and YOLOv7**^+^**-88%, which represent the changing process of pruning performance indexes of YOLOv7 and YOLOv7**^+^**, respectively. As can be seen from Figure 8, the parameters of YOLOv7 and YOLOv7**^+^** change in the pruning process. With the increase in the pruning rate, the reasoning speed of YOLOv7 and YOLOv7**^+^** image recognition continuously increases, while other parameters all decrease at a certain speed. The ultimate purpose of this experiment is to improve the reasoning speed of the detection algorithm as far as possible on the premise of improving the accuracy of object detection. It can be seen from Figure 8 that the mAP performance of YOLOv7**^+^**-86% and YOLOv7**^+^**-87% is optimal. The FPS of the YOLOv7**^+^**-87% model is 14 higher than that of the YOLOv7**^+^**-86% model when other evaluation indicators are essentially the same. Therefore, YOLOv7**^+^**-87% was selected as the final model for optimization. Compared with the original YOLOv7 model, in which the input image for network settings is 640 × 640, the trainable parameters of the YOLOv7**^+^**-87% model are reduced by 97.66%, the model space is reduced by 96.36%, and the reasoning speed is increased by 432.30%. The mAP of the optimized YOLOv7**^+^**-87% is increased by 9.06% compared with the original mAP of YOLOv7. The comparison of the above data confirms the effectiveness of the optimization strategy.

**Figure 9:**
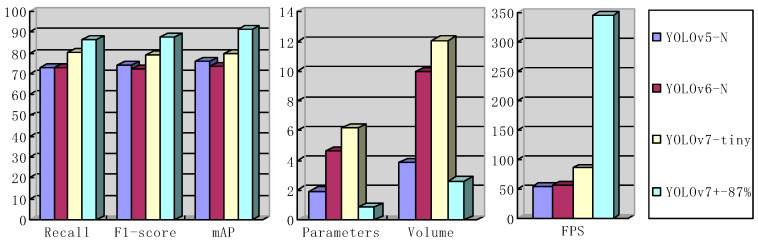
Evaluation indexes of network YOLOv5-N, YOLOv6-N, YOLOv7-Tiny, and YOLOv7**^+^**-87% when the size of network training image is 864 × 864.

The performance of YOLOv7**^+^**-87% is compared with that of existing lightweight algorithms: Table 4 lists the most representative lightweight network performance indicators. These algorithms are all trained by default under the same environment of the original author’s network configuration. By comparing the evaluation indexes of YOLOv5-N, YOLOv6-N, YOLOv7-Tiny, and YOLOv7**^+^**-87% in Figure 9, an appropriate basic network model is selected. As can be seen from Figure 9, the network model of YOLOv7**^+^**-87% shows the optimal performance compared with the network model of YOLOv5-N, YOLOv6-N, and YOLOv7-tiny when considering the evaluation indicators Recall, F1-score, and mAP. The parameters and volume of the YOLOv7**^+^**-87% network model are also reduced to the minimum parameter size, saving space for deployment to hardware devices. The operation speed of its inference picture also shows outstanding operation efficiency in YOLOv5-N, YOLOv6-N, and YOLOv7-tiny. According to the comparison in Figure 9, the evaluation index of training of the YOLOv7**^+^**-87% network model on the BDD100K dataset is generally better than that of other network models. Therefore, YOLOv7**^+^**-87% was selected as the object detection model of this experiment.

In order to improve the detection of objects on foggy traffic roads, this experiment selects the dark channel de-fogging algorithm to process the foggy environment. The advantages of this algorithm are the good restoration effect and simple application scenario, while the disadvantage is the low operating efficiency of the algorithm. In order to better meet the real-time nature of traffic roads, we first take the original picture with fog by Gaussian Smoothing and then down-sample it 4 times. Then, dark channel defogging is carried out on the image after downsampling. In this case, the de-fogging efficiency has been effectively improved. Then, the image defogging is up-sampled, and then Gaussian Smoothing is performed after up-sampling. The final image obtained meets our requirement of resolution. Then it is combined with the YOLOv7**^+^**-87% lightweight object detection model to meet the real-time detection requirements of foggy traffic roads.

**Figure 10:**
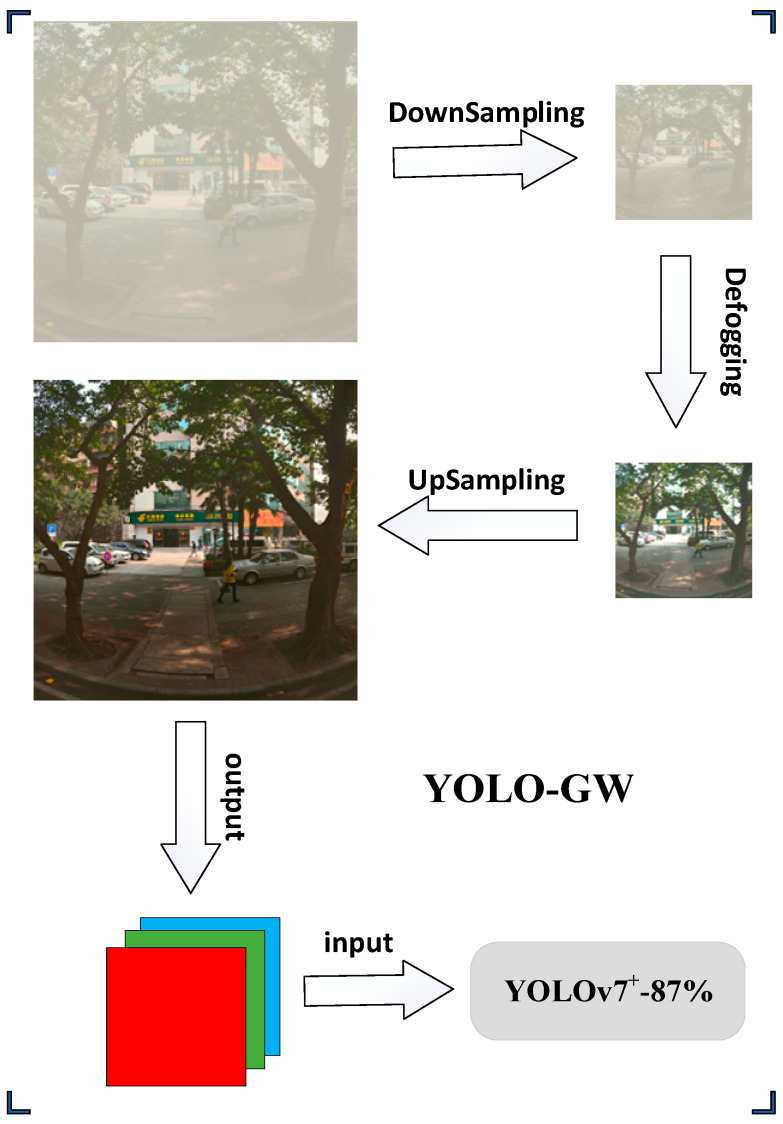
Structure of YOLO-GW object detection algorithm.

In order to verify the effectiveness of the fog detection algorithm, several excellent object detection algorithms, namely YOLOv5-N, YOLOv6-N, YOLOv7-Tiny, YOLOv7, YOLOv7**^+^**-87%, and YOLO-GW, were selected for visual comparison in this experiment to prove the effectiveness of the fog detection algorithm. Among them, YOLO-GW adds a de-fogging strategy on the basis of the YOLOv7**^+^**-87% algorithm to improve pedestrian detection in a foggy traffic environment (the YOLO-GW algorithm model adds a defogging strategy, while other comparison algorithm models do not add a defogging strategy in the detection process). The de-fogging algorithm strategy does not participate in model training but rather only shows a de-fogging effect in the image detection process. As shown in Table 5, YOLOv7**^+^**-87% and YOLO-GW target detection algorithms have the same network parameters and volume. Compared with the parameters and volume of the original network YOLOv7, the trainable parameters of the YOLOv7**^+^**-87% model are reduced by 97.66% and the model space is reduced by 96.36%, making it possible for the model to be deployed on the chip. The experiment was conducted in the same hardware environment configuration. The YOLO-GW algorithm in the table is based on the YOLOv7**^+^**-87% algorithm, which adds the algorithm model of de-fogging algorithm strategy optimization. As shown in Figure 10, it is equivalent to each frame image input into the network model for classification after the de-fogging strategy is carried out, and the detection accuracy of this model does not change. Due to the addition of the de-fogging strategy, the FPS of the YOLOv7**^+^**-87% algorithm is reduced by 240, but it shows an excellent detection effect when detecting a foggy environment. Compared with the YOLOv5-N, YOLOv6-N, YOLOv7-tiny, and YOLOv7 target detection algorithms, the mAP of YOLO-GW shows the best performance.

In order to further verify the effectiveness of the YOLO-GW object detection algorithm, images of the original sunny day, mild foggy day, moderate foggy day, and severe foggy day are selected for visual detection and verification. The verified images are video data taken from actual road conditions, while the original images are from sunny days. In order to compare the detection effect of the YOLO-GW target detection algorithm with that of YOLOv5-N, YOLOv6-N, YOLOv7-tiny, and YOLOv7 in mild, moderate, and severe foggy days, in this experiment, the effects of mild fog, moderate fog, and severe fog were added to the selected sunny day pictures. The data are used to perform visual detection and comparison of YOLOv5-N, YOLOv6-N, YOLOv7-tiny, YOLOv7, and YOLO-GW, as shown in the figure below.

**Figure 11:**
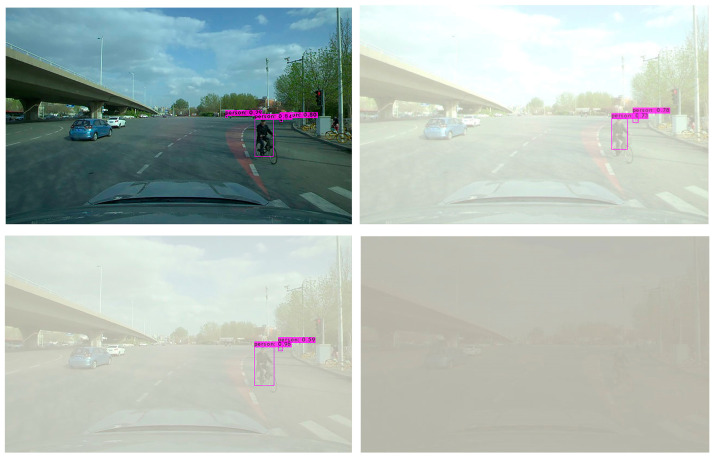
The detection effect of YOLOv5-N target detection algorithm in sunny, mildly foggy, moderately foggy, and severely foggy environments, respectively.

**Figure 12:**
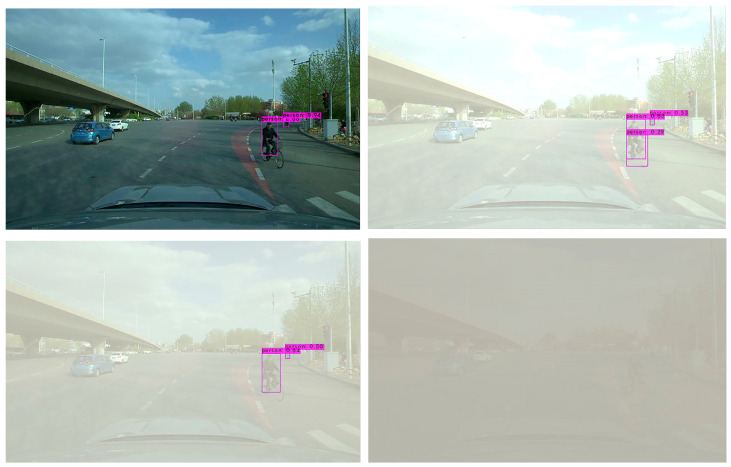
The detection effect of YOLOv6-N target detection algorithm in sunny, mildly foggy, moderately foggy, and severely foggy environments, respectively.

**Figure 13:**
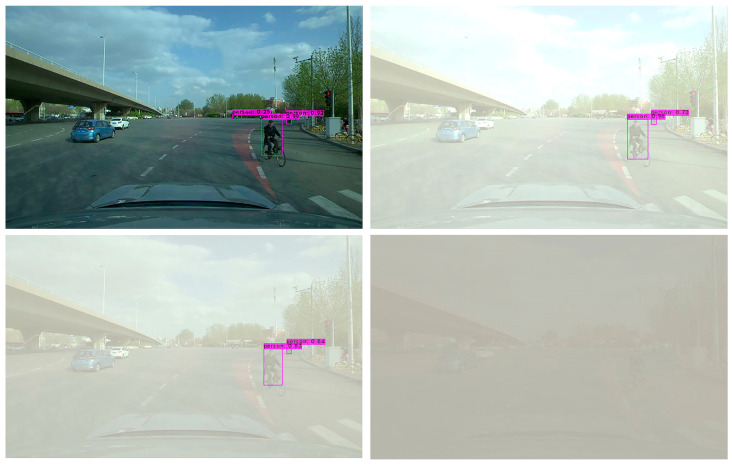
The detection effect of YOLOv7-tiny target detection algorithm in sunny, mildly foggy, moderately foggy, and severely foggy environments, respectively.

**Figure 14:**
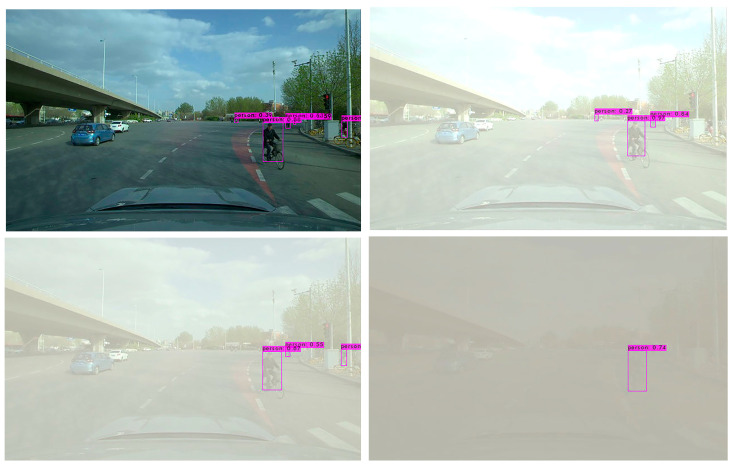
The detection effect of YOLOv7 target detection algorithm in sunny, mildly foggy, moderately foggy, and severely foggy environments, respectively.

**Figure 15:**
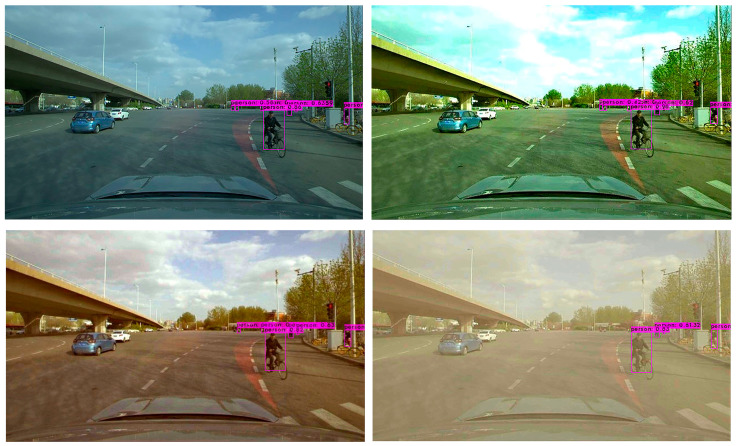
The detection effect of YOLO-GW target detection algorithm in sunny, mildly foggy, moderately foggy, and severely foggy environments, respectively.

Visualization effect analysis: It can be seen from the detection of clear day images in Figure 11, Figure 12 and Figure 13 that although YOLOv5-N, YOLOv6-N, and YOLOv7-tiny have fast reasoning speed, they are not ideal in visual performance detection, and the situation of missing detection is relatively serious. With the increase in fog concentration, there will be false detection. When the fog concentration is heavy, YOLOv5-N, YOLOv6-N, and YOLOv7-tiny cannot detect pedestrians in the environment. As can be seen from Figure 14, YOLOv7 has a better object detection effect than YOLOv5-N, YOLOv6-N, and YOLOv7-Tiny. However, with the deepening of fog concentration, YOLOv7′s detection effect gradually weakens, and only nearby pedestrians can be detected when the environment is thick fog. As can be seen from Figure 15, compared with YOLOv5-N, YOLOv6-N, YOLOv7-Tiny, and YOLOv7, YOLO-GW showed a better detection effect in visual detection and verification of images in sunny, mildly foggy, moderately foggy, or severely foggy days. Therefore, YOLO-GW is more suitable for pedestrian detection in a foggy environment compared with YOLOv5-N, YOLOv6-N, YOLOv7-Tiny, and YOLOv7 target detection algorithms.

## 6. Conclusions

In this paper, an algorithm named YOLO-GW was proposed for real-time pedestrian detection in a foggy environment. In order to realize the object detection algorithm’s detection of pedestrians in a foggy environment, the dark channel de-fogging algorithm is added, and four layers of down-sampling and up-sampling modules are added to improve the de-fogging speed. In order to ensure the detection accuracy of the object detection algorithm in a complex environment, the ECA module and a detection head were added to the network architecture of YOLOv7 in this experiment to improve object classification and regression, and an 864 × 864 network input size was used for model training. Finally, we combined the pruning algorithm to optimize the training model to obtain the YOLO-GW detection algorithm. Compared to YOLOv7, YOLO-GW increased FPS by 63.08%, mAP increased by 9.06%, parameters decreased by 97.66%, and volume decreased by 96.36%. Smaller training parameters and model space make it possible for the YOLO-GW target detection algorithm to be deployed on the chip. According to the comparison of visualization effects in Figure 14 and Figure 15, YOLO-GW is more suitable for pedestrian detection in a foggy environment than YOLOv7.

## Figures and Tables

**Table 1:** Experimental environment configuration.

Name	Related Configuration
CPU set/GHZ	Inter Xeon E5-2603
GPU	Geforce RTX 2080 Super
GPU Accelerate library	CUDA11.7, CUDNN8.6.0
Deep learning framework	Pytorch 1.13.1
Operating system	Ubuntu 20.04

**Table 2:** Representative experimental results of YOLOv7. It is primarily used to compare the network performance of the original YOLOv7 network model with the ECA module or detection head added to the original YOLOv7 network and the ECA module and detection head added to the original YOLOv7 network. To verify the rationality of adding ECA module and detection head to the original network model of YOLOv7 in this experiment.

Model	Add Model	Input size	Recall (%)	F1-Score (%)	mAP (%)	FPS	Parameters (M)	Volume (MB)
YOLOv7	/	640	83.70	82.20	83.90	65	36.90	72.10
		864	87.80	87.90	86.10	27		
YOLOv7	Eca	640	84.98	83.90	84.20	63	38.70	73.39
		864	89.10	90.10	89.70	24		
YOLOv7	Detection head	640	85.69	84.79	85.98	62	39.90	74.69
		864	91.78	91.69	93.10	24		
YOLOv7	Eca + Detection head	640	86.76	85.90	86.71	59	42.70	76.12
		864	92.98	93.21	94.50	22		

**Table 3:** Performance comparison between YOLOv7 and YOLOv7**^+^** after different pruning. In order to distinguish the pruning algorithm of YOLOv7 original network from that of YOLOv7 optimized by ECA module and Detection head, the pruning algorithm of YOLOv7 original network was represented by YOLOv7*. The YOLOv7 pruning algorithm optimized by ECA module and Detection head was represented by YOLOv7**^+^**.

Model	Input Size	Recall (%)	F1-Score (%)	mAP (%)	FPS	Parameters (M)	Volume (MB)
YOLOv7	640	83.70	82.20	83.90	65	36.90	72.10
	864	87.80	87.90	86.10	27		
YOLOv7*-50%	640	79.50	82.00	81.20	167	6.90	16.70
	864	86.10	84.80	83.70	76	8.61	18.60
YOLOv7*-86%	864	85.60	82.30	83.20	341	0.99	2.70
YOLOv7*-87%	864	85.40	82.20	83.10	356	0.71	2.20
YOLOv7*-88%	864	82.20	81.10	82.50	367	0.32	1.50
YOLOv7^+^	640	86.76	85.90	86.71	59	42.70	76.12
	864	92.98	93.21	94.50	22		
YOLOv7^+^-50%	640	84.80	83.00	85.10	152	7.78	18.19
	864	92.50	90.00	92.60	67	9.18	20.80
YOLOv7+-86%	864	89.90	89.10	91.98	332	1.18	3.29
YOLOv7+-87%	864	86.40	87.70	91.50	346	0.86	2.62
YOLOv7+-88%	864	78.90	81.50	83.70	357	0.34	1.66

**Table 4:** Comparison of experimental results between YOLOv5-N, YOLOv6-N, and YOLOv7-tiny lightweight networks and pruning network YOLOv7**^+^**-87%.

Model	Input Size	Recall (%)	F1-Score (%)	mAP (%)	FPS	Parameters (M)	Volume (MB)
YOLOv5-N	640	71.10	72.30	71.09	159	1.90	3.87
	864	73.10	74.40	75.90	54		
YOLOv6-N	640	70.60	69.40	70.40	166	4.65	9.97
	864	72.90	72.60	73.50	57		
YOLOv7-tiny	640	73.70	71.80	73.40	226	6.20	12.10
	864	80.40	79.10	79.60	86		
YOLOv7^+^-87%	864	86.40	87.70	91.50	346	0.86	2.62

**Table 5:** Performance indicators of object detection algorithm (YOLO-GW algorithm model added a defogging strategy in the detection process, while YOLOv5-N, YOLOv6-N, YOLOv7-tiny, YOLOv7, and YOLOv7**^+^**-87% algorithm models did not add a defogging strategy in the detection process).

Model	Input Size	mAP	FPS	Parameters (M)	Volume (MB)
YOLOv5-N	640	71.09	159	1.90	3.87
YOLOv6-N	640	70.40	166	4.56	9.97
YOLOv7-tiny	640	73.40	226	6.20	12.10
YOLOv7	640	83.90	65	36.90	72.10
YOLOv7^+^-87%	864	91.50	346	0.86	2.62
YOLO-GW	864	91.50	106	0.86	2.62

## Data Availability

Data available in a publicly accessible repository. The data presented in this study are openly available in [repository name Berkeley DeepDrive] at [https://bdd-data.berkeley.edu/portal.html#download], reference number [19].
