# Correction: Liu, X.; Lin, Y. YOLO-GW: Quickly and Accurately Detecting Pedestrians in a Foggy Traffic Environment. *Sensors* 2023, *23*, 5539

**DOI:** 10.3390/s26113277

**Published:** 2026-05-22

**Authors:** Xinchao Liu, Yier Lin

**Affiliations:** College of Mechanical Engineering, Tianjin University of Science and Technology, Tianjin 300222, China; liuxinchao@mail.tust.edu.cn

## Error in Figure

In the original publication [1], there was a mistake in Figure 7 as published. The label “mAP (864 × 864)” has been input as “mAP (846 × 846)” by mistake. The corrected Figure 7 appears below.

There is a typo in Figure 2 Caption. The “YOLOV7*” has been updated to “YOLOv7*”.

## Text Correction

There were errors in the original publication.

1. In Section 1, Paragraph 4. The sentence “Compared with the original model YOLOv7, the trainable parameters of model YOLOv7^+^-87% are reduced by 97.66%, the model space is reduced by 96.36%, and the reasoning speed is 423.30% faster.” has been updated to “Compared with the original model YOLOv7, the trainable parameters of model YOLOv7^+^-87% are reduced by 97.66%, the model space is reduced by 96.36%, and the reasoning speed is 432.30% faster.”

2. In Section 4.2. The sentence “Furthermore, 10,000 images containing pedestrians are selected as the training set of this experiment. The verification set and test set adopt 1000 self-made datasets, which are 640 × 400-pixel pictures, as shown in Figure 6.” has been updated to “Furthermore, 10,000 pedestrian-containing images are selected from all available images to form the experimental dataset. Specifically, the training set comprises 7000 pedestrian images, the validation set comprises 1500 pedestrian images, and the test set comprises 1500 pedestrian images. For visualization validation, 1000 self-constructed dataset images (which are not involved in training) are adopted. These images are of 640 × 400 pixels, as shown in Figure 6.”

3. In Section 4.3, Paragraph 1. The sentence “Both YOLOv7 and YOLO-GW were trained with a batch gradient descent.” has been updated to “Both YOLOv7 and YOLOv7^+^-87% were trained with a batch gradient descent.”

4. In Section 5, Paragraph 2. The sentence “In order to further improve the algorithm performance of YOLOv7, we changed the network structure of YOLOv7 with three detection heads into a network structure of four detection heads and carried out 846 × 846 large-scale training to further strengthen object classification and regression, as well as improve the effective feature extraction.” has been updated to “In order to further improve the algorithm performance of YOLOv7, we changed the network structure of YOLOv7 with three detection heads into a network structure of four detection heads and carried out 864 × 864 large-scale training to further strengthen object classification and regression, as well as improve the effective feature extraction.”

5. In Section 5, Paragraph 3. The sentence “The FPS of the YOLOv7**^+^**-87% model is 14 times higher than that of the YOLOv7**^+^**-86% model when other evaluation indicators are essentially the same.” has been updated to “The FPS of the YOLOv7**^+^**-87% model is 14 higher than that of the YOLOv7**^+^**-86% model when other evaluation indicators are essentially the same.” The sentences “Compared with the original YOLOv7 model, in which the input image for network settings is 640 × 640, the trainable parameters of the YOLOv7**^+^**-87% model are reduced by 97.66%, the model space is reduced by 96.36%, and the reasoning speed is increased by 423.30%. The mAP of the optimized YOLOv7**^+^**-87% is increased by 9% compared with the original mAP of YOLOv7.” have been updated to “Compared with the original YOLOv7 model, in which the input image for network settings is 640 × 640, the trainable parameters of the YOLOv7**^+^**-87% model are reduced by 97.66%, the model space is reduced by 96.36%, and the reasoning speed is increased by 432.30%. The mAP of the optimized YOLOv7**^+^**-87% is increased by 9.06% compared with the original mAP of YOLOv7.”

The authors state that the scientific conclusions are unaffected. This correction was approved by the Academic Editor. The original publication has also been updated.

## Figures and Tables

**Figure 7:**
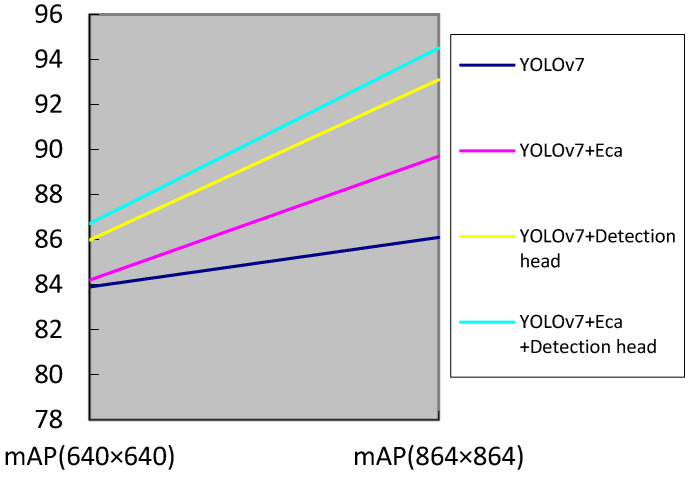
Comparison of mAP performance of ECA module and detection head module was added to YOLOv7 algorithm model.

## References

[1] Liu X., Lin Y. (2023). YOLO-GW: Quickly and Accurately Detecting Pedestrians in a Foggy Traffic Environment. Sensors.

